# Online public discourse on artificial intelligence and ethics in China: context, content, and implications

**DOI:** 10.1007/s00146-021-01309-7

**Published:** 2021-11-16

**Authors:** Yishu Mao, Kristin Shi-Kupfer

**Affiliations:** 1 Lise Meitner Research Group “China in the Global System of Science”, Max Planck Institute for the History of Science, Berlin, Germany; 2 Contemporary China Studies, Universität Trier, Trier, Germany

**Keywords:** Artificial intelligence, China, Ethics, Governance, Social media, Public opinion, Content analysis

## Abstract

The societal and ethical implications of artificial intelligence (AI) have sparked discussions among academics, policymakers and the public around the world. What has gone unnoticed so far are the likewise vibrant discussions in China. We analyzed a large sample of discussions about AI ethics on two Chinese social media platforms. Findings suggest that participants were diverse, and included scholars, IT industry actors, journalists, and members of the general public. They addressed a broad range of concerns associated with the application of AI in various fields. Some even gave recommendations on how to tackle these issues. We argue that these discussions are a valuable source for understanding the future trajectory of AI development in China as well as implications for global dialogue on AI governance.

## Introduction

“When the young people of Generation Z start discussing AI ethics, constructing a set of perfect rules for AI governance is no longer out of reach.” So claimed Alter, the author of a widely read and reposted article published on Chinese social media WeChat (2020). The post referred to a popular vlog produced by a journalism student from Tsinghua University on the video sharing site BiliBili who interviewed two young researchers from China’s top AI start-up Megvii about the ethical issues around AI (Xiaosu 2020). Although Alter’s expectations for “perfect” AI governance rules may be overly optimistic, it is worth noting that the rapid development and deployment of AI in China has been accompanied by growing societal discussions about its social and ethical implications. Social media platforms have become important fora for multi-stakeholder exchanges on fundamental questions, hopes, concerns and recommendations on how AI should be developed, used and regulated.

How AI is represented, communicated and perceived in cultural narratives can profoundly influence research, reception and regulation (Cave et al. 2018). Existing analyses of public communication about AI, by means of English-language media content analysis, have found that media coverage tends to focus on its economic impact, despite the recently growing attention to related ethical issues (Chuan et al. 2019; Ouchchy et al. 2020). Moreover, surveys among the general public found skewed perceptions that are either utopian or dystopian. Lack of awareness regarding the realistic implications of AI is a significant hurdle in its uptake for social benefit (Cave et al. 2019).

China’s emergence as a global leader in the field of AI raises the importance of understanding its development trajectory in this specific cultural context. The government’s ambitious AI strategy, regulatory approaches, the vast resources invested, and the capabilities of Chinese AI players are important factors shaping this trajectory (Allen 2019; Sheehan 2019; Roberts et al. 2021; Colvin et al. 2020; Ding 2018). However, despite the party-state’s control over the public sphere, societal discourse in China can shed light on what sociotechnical future the population of over one billion can imagine and is currently negotiating (Jasanoff and Kim 2015). With the aim of examining online public discourse on ethical issues around AI in China, the authors focused on the following questions:

1. How are the ethical and societal implications of AI being discussed?
2. Who is shaping the discussions?
3. What are the similarities and differences between the opinions of different stakeholders?
4. What are the implications of Chinese public discourse for global dialogue in AI governance?

With this focus we sought to provide insights into the spectrum of opinions and therefore a more holistic understanding of China’s approach to AI. The paper is structured as follows. Section 2 gives an overview of the context of social media discussions on AI ethics as well as an introduction of the two platforms from which we collected the data for our analysis. The research methodology and analytical approach is outlined in Sect. 3. The results of the content analysis are discussed in Sect. 4. The paper concludes with implications for further research, also regarding dialogue on AI governance at the international level.

## The context: social media play an important role in political and science communication

The Chinese government is aiming to establish an ethical framework and a system of laws and regulations to govern AI by 2030 (State Council 2017). Several high-level ethical guidelines such as the Beijing AI Principles and the Governance Principles for a New Generation of AI have been released as collaborative efforts between government-affiliated research institutions, universities, and leading companies. However, translating lofty principles into meaningful moral axioms that industry and social actors can relate to requires public engagement. AI-related science popularization activities, as well as public opinion guidance, were also listed as key tasks to be completed in the 2017 New Generation AI Development Plan (State Council 2017).

Social media platforms play an increasingly important role in mediating China’s public sphere as the result of rapid adoption of the Internet and smartphones. However, the role these platforms play is by no means simply as channels for unrestricted public opinion. Besides censorship, social media content is often subjected to overt propaganda or covert efforts to guide opinion (Tai and Fu 2020; Creemers 2017). The latter are found to be done by both hired “internet commentators” or “50 cent party members”^1^ and public intellectuals (King et al. 2017; Abb 2021). In the case of discourse relating to AI in general, and not any specific ethical aspects, researchers have found that social media content largely conforms to the party-state media’s framing of AI’s economic benefits, with little evidence of critical debates (Zeng et al. 2020).

Nevertheless, social media platforms can facilitate online public opinion and remain as places where diverse ideologies and voices relating to current social and political affairs can be found (Stockmann and Luo 2017; Shi-Kupfer et al. 2017; Shi-Kupfer and Mao 2020). In some cases, these platforms help to form important networks and spaces for citizens to collectively voice their interests and concerns, and to facilitate activism (Shan and Tang 2017; Mao 2020; Gleiss 2015; Lei 2018). There is ample evidence that opinions and concerns voiced on social media can set the agenda for traditional (although not party-state) media and can gain the recognition and response from the government (Wang [Bibr CR60][Bibr CR61]; Luo 2014). Moreover, online criticism has also frequently exposed failings of commercial actors and exerted pressure on the government to hold them accountable. For example, in 2018 the Chinese AI startup Megvii was widely criticized online for a demo video of its facial and emotion surveillance system designed for schools, which was posted on the social media platform Weibo. In the wake of this backlash, the Ministry of Education issued “Opinions on Guiding and Standardizing the Orderly and Healthy Development of Mobile Internet Applications in Education” and stated the intention of regulating AI applications for educational use (Ministry of Education of the People’s Republic of China 2019; Wu 2019).

Social media platforms are also becoming increasingly important for science communication in China. The government encouraged the scientific community to use social media as channels for disseminating scientific knowledge among the public and to encourage public engagement with science (Xinhua 2019; Dijkstra and Yin 2019). Partly validating this strategy, researchers found that information on social media can influence people’s attitudes toward and support for controversial technologies such as genetically modified organisms (GMO) and AI (You 2019; Cui and Wu 2021).

The role of social media platforms in China’s political and science communication make them a valuable source for analyzing public discourse on AI. However, there are multiple platforms with different technical attributes and user bases which perform these roles in different ways and to a different extent. Our study focuses on two platforms—WeChat and Zhihu—because of their shared popularity and yet distinctively different communication styles, user bases and content foci.

WeChat, a “super app” that combines the function of Whatsapp and Facebook and even integrates other commercial and public services is the most popular social media platform in China. In 2020, WeChat’s active monthly users in China numbered over 1.2 billion. It is equally popular among men and women, and its users cover all age groups (58.5% of the users are 30 or below. 41.5% of the users are above 30 (Ho 2021). The app’s public account function, which we focused on in our research, allows individuals and organizations to publish articles to their subscribers with text, pictures, audios and videos. These articles may be short but can also be the length of an entire academic paper. There are around 8.5 million public accounts, many of them having several million subscribers and they cover a variety of topics (Li [Bibr CR33][Bibr CR35]). Some of these account owners are prominent figures and organizations in various fields offline. The communication style and users of WeChat public accounts mostly represent the cultural elites who use the platform to promote new concepts and to institutionalize idea.

The second platform, Zhihu, is a Chinese Q&A online forum similar to Quora. As of 2019 it had 220 million registered users and more than 130 million user-generated answers (Smith 2021). Established with the slogan “Let people better share knowledge, experience and insights and find their own answers,” the market strength of the platform is its peer-to-peer knowledge network. It is deemed as the best Chinese social media platform to get professional expert insights on various topics (Graziani 2018). Although most Zhihu users do not give details of their personal identity, professions and fields of expertise are commonly used as identity markers. Analysis of Zhihu’s user base has identified that most users are professionals in the tech industry, students of computer sciences, and other highly educated groups (Cxb168 2019; Xueshenkeji 2020). The gender imbalance in these fields also seems to be reflected by the dominant presence of male users (53% male, 33% female and 14% unknown) (Zhang 2019). Content from Zhihu mainly represents individual opinions from the frontier of technological development in China.

To sum up, content on social media can provide valuable material for analyzing online public discourse on the social and ethical implications of AI, whether it be part of the official opinion-guiding efforts, Chinese cultural elites’ genuine intellectual investigations, or societal discussions. Our choices of two distinctive social media platforms, WeChat and Zhihu, largely cover the potentially different dimensions of public discourse.

## Methods

To understand the discussions about AI ethics on Chinese social media in a structured and contextualized way, we adopted qualitative content analysis methods in our study. As a social studies research technique commonly used in communication studies, content analysis applies systematic and rule-guided procedures to the analysis of large amounts of texts such as media content. This includes developing a category system instrumental in understanding the texts, using the system to encode texts, and generating various analyses such as quantitative category occurrences and qualitative contexts. The main goal of this technique is to reduce text materials in such a way that the essential contents remain and prominent themes emerge (Mayring 2014). This methodology has been frequently used to understand the meanings a group of people or a culture attribute to an issue within a specific context, taking that meaning can be derived from content of communication, which supports inference about its social context (Krippendorff 1989). It is therefore the most suitable for our study which seeks to navigate through emerging online discussions in China on AI ethics.

### Data collection

WeChat’s public application programming interface (API) is notoriously difficult to access. To collect relevant posts from its public accounts, we used the proven method of web-scraping through the third-party search engine Sogou, which provides access to historical data of WeChat public accounts (You et al. 2018; Zeng et al. 2020). When we started our research, the search engine Sogou did not support site search for Zhihu posts, so we used another Chinese search engine, Baidu, to collect Zhihu data. However, in a later phase when we were updating our database, Sogou added this function. Once it was technically possible, we used Sogou to double-check the results and added the new results that had not been yielded by Baidu.

The keyword combinations “AI Ethics” and “AI Morality”^2^ were used separately to retrieve data from the time range set from the earliest post up to and including December 31, 2020. The results from WeChat included a total of 1173 posts with the first post on record from November 24, 2014. After removing duplicates, event/book promotions, articles that only contain audio, video, and image content and, for the purpose of our analysis, excluding non-opinion pieces such as purely factual news reports, 395 posts were qualified for further analysis. The results from Zhihu included 1123^3^ threads (one question with three answers) with the first on record from November 28, 2014.^4^ After removing duplicates, threads with less than three answers, question posts composed of only one sentence, and answers which are too short or irrelevant, 372 posts (124 questions with 3 answers each) were included in the final analysis.

### Data analysis

To perform content analysis on the dataset we collected, we first developed a coding scheme based on a literature review of global discussions on AI ethics. We also built upon the concept of framing in media and communication studies, which is defined as selecting “some aspects of a perceived reality” to enhance their salience “in such a way as to promote a particular problem definition, causal interpretation, moral evaluation, and/or treatment recommendation” (Entman 1993, p. 53). According to the coding scheme, we treated an article on WeChat and a question or answer on Zhihu as a unit of analysis and coded the following variables: author type,^5^ foreign sources(s) of reference, context(s) of discussion, risk/opportunity/neutral assessment/not applicable, reasons given for above assessment,^6^ and recommendation(s).^7^

The two authors independently conducted manual coding on the data from each platform. To ensure intercoder reliability, we used 20 random listings from each platform as a sample to code, compared the results with each other, and reached an agreement. Then the two authors independently conducted manual coding on data collected from each of the two platforms, respectively.

### Limitations

We acknowledge several limitations of our research. First, our data collection was highly reliant on the search engines we used, and we cannot assess how the underlying algorithms could have influenced the search results. To avoid personalized results, we conducted a search for WeChat articles on Sogou with a newly registered account without any previous data usage. The searches for Zhihu posts were conducted on Baidu and later on Sogou without a personal Zhihu login. However, this does not ensure access to the complete and unbiased pool of relevant data. Second, although the user demographics of WeChat and Zhihu are diverse, it should be noted that the data from two specific social media platforms can only include the discourse and views of those who have access to digital technologies and use them. Third, our use of the keyword “AI” rather than specific terms such as “facial recognition,” or “content recommendation algorithms” inevitably missed some of the discussions on specific AI applications, which is worthy of further research.

## Results and discussions

### Types of authors

**Fig. 1:**
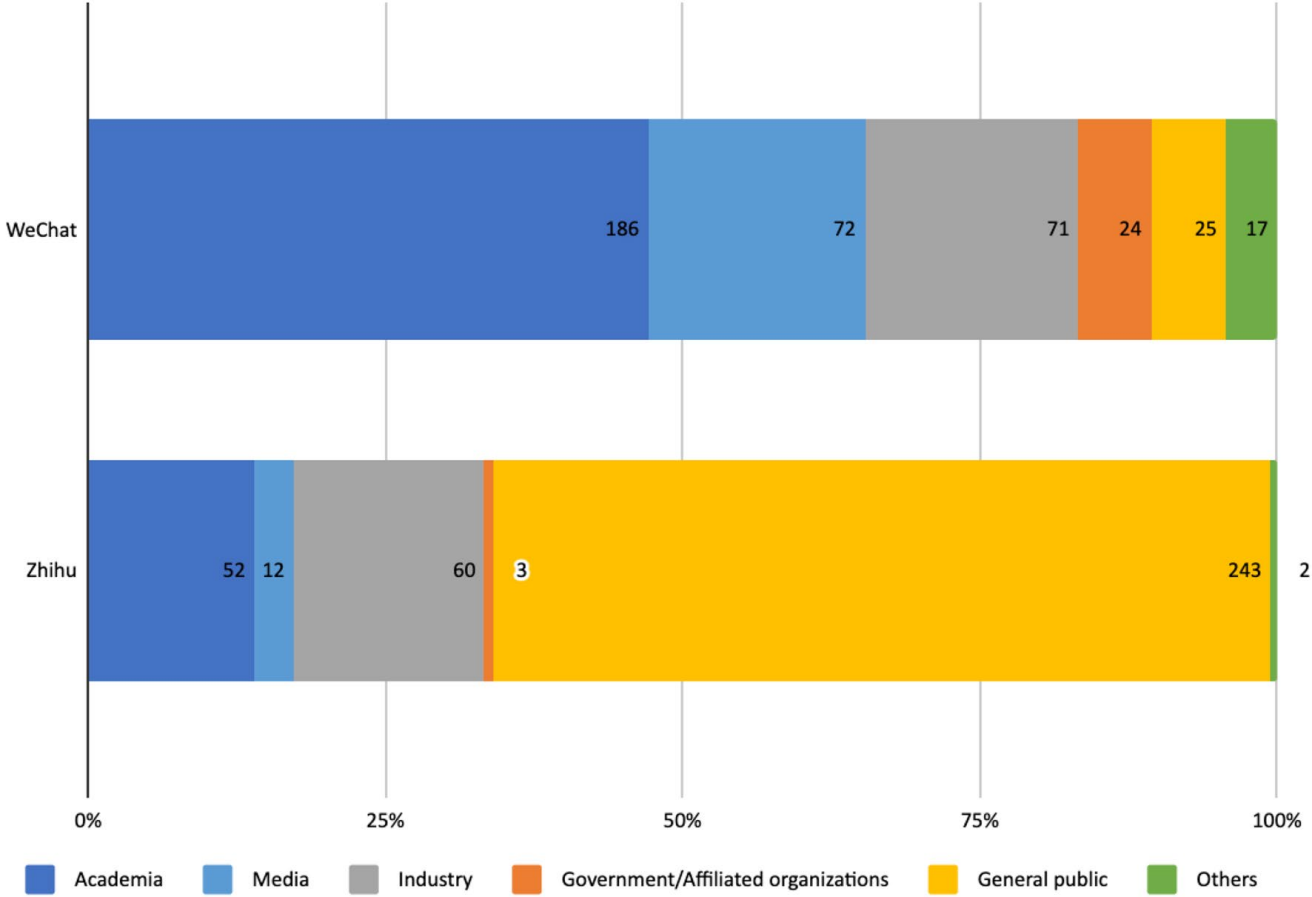
Types of authors from analyzed articles on WeChat and Zhihu

On WeChat, 47.1% of the analyzed articles’ authors were researchers in the field of AI ethics and governance. The most prolific include Duan Weiwen, director for the Department of Philosophy of Science and Technology in the Institute of Philosophy at the Chinese Academy of Social Sciences, Zeng Yi, director for the Research Center on AI Ethics and Safety, Beijing Academy of Artificial Intelligence, and Chen Xiaoping, director of Robotics Lab at University of Science and Technology of China. The public accounts of media outlets, especially those focusing on the tech industry such as Leiphone.com and Jiqizhixin.com, published a further 18.2% of the articles. Individuals and organizations in the tech industry accounted for 18% of the articles. Among them, Tencent Research Institute, the social research arm of the Chinese internet giant, is the most prolific account among all types of authors. Since 2017 it has published numerous articles about potential ethical issues associated with AI, introducing global and especially European ethics principles and regulations aimed at the Chinese public. Among the remaining articles, 6.3% were published by members of the general public, who were identified as such by the account type ‘individual’ on the accounts’ ‘about’ pages. 6.1% were published by government affiliated organizations and party-state media. The remaining 4.3% were published by organizations categorized as ‘Others.’ These included local associations promoting science and technology, religious associations and non-governmental organizations (NGOs). Notably, only one Chinese environmental NGO and no NGO with specific focus on AI governance was identified in the discussions on WeChat (Fig. 1).

On Zhihu, authors from the general public accounted for 65.3% of the posts. The second largest group of contributors (16.1%) were authors with a self-stated industry background. They were mostly individuals, but featured were also institutional accounts of companies broadly related to the field of AI such as Microsoft Asia, Amazon, iFlytek. Like on WeChat, Tencent Research Institute was one of the most active Chinese institutional accounts here, offering substantial analysis and links to further studies. Users with academic backgrounds accounted for 14% of the posts. Media accounts (3.2%) and authors with government or government-affiliated background (0.8%) were nearly absent—at least based on the publicly displayed information.

Analysis showed that a wide range of stakeholders participated in the AI ethics discussions on both platforms. As WeChat public accounts and Zhihu were chosen for this study due to the different characteristics of their user base, our analysis of the type of authors proved that the prominent voices on the two platforms were indeed quite different. The majority of authors on WeChat were from academia, the media or industry, representing cultural elites, while on Zhihu most were members of the general public and individual IT professionals. This difference provided the background for analyzing how and why opinions expressed on the two platforms differ, which we elaborate on in the subsequent sections.

### Foreign references

**Fig. 2:**
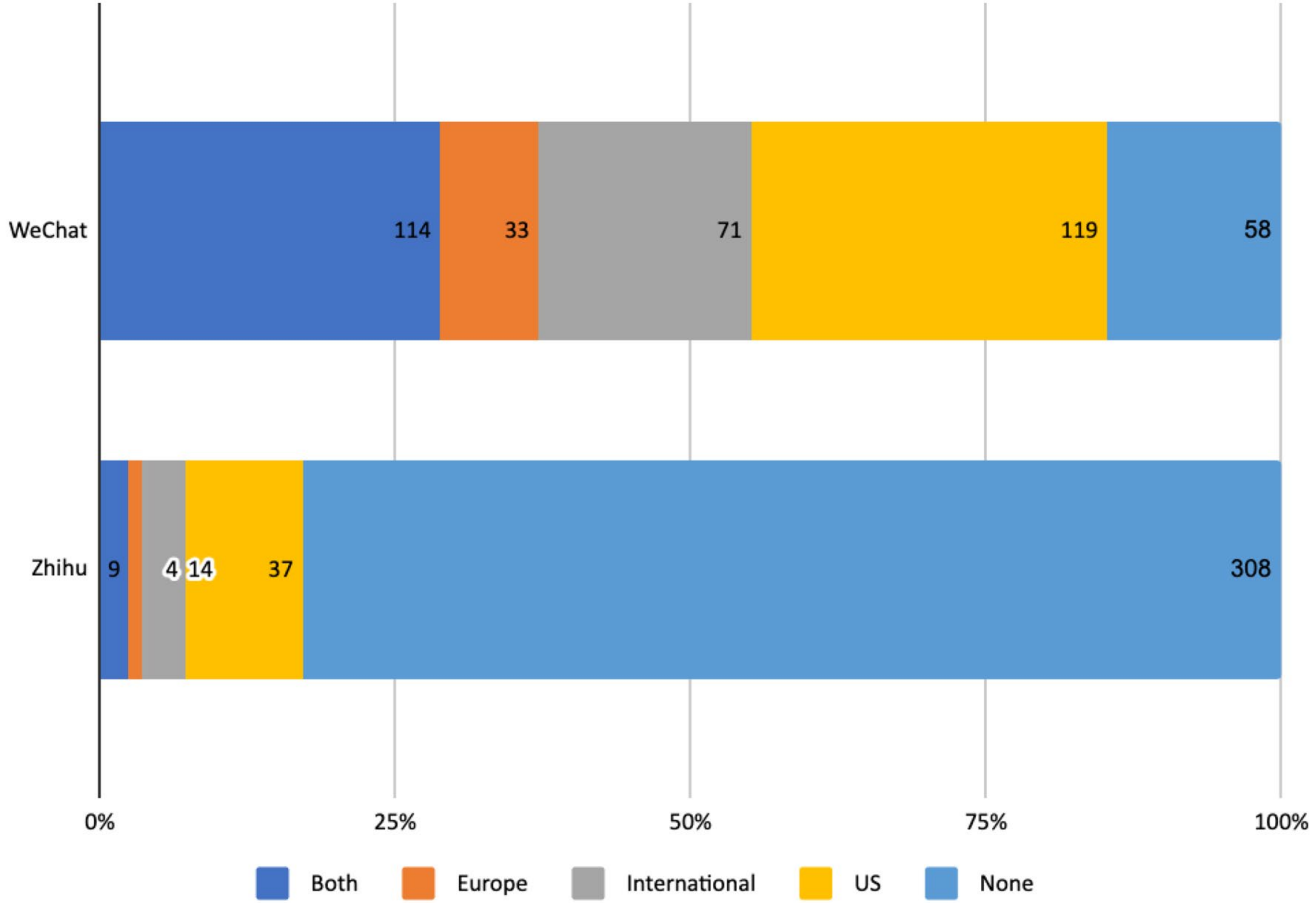
Use of foreign references on WeChat and Zhihu

To discover how public discourse on AI ethics in China relates to the global discourse, we coded the sources of foreign references when they were used. On WeChat, 30.1% of the authors cited one or more sources from the US, 8.4% from Europe, and 28.9% from both Europe and the USA, while 18% cited sources from other parts of the world. The most frequently cited set of principles is the “Three Laws of Robotics” by the science fiction writer Isaac Asimov. The EU commission’s “Ethics guidelines for trustworthy AI,” Microsoft’s “Responsible AI principles,” and the IEEE’s *Ethically Aligned Design* were also frequently cited. Notably, in multiple academic papers posted on WeChat, there were lengthy interpretations of the EU's “Ethics guidelines for trustworthy AI” and even discussions of its potential implementation in specific fields such as education and healthcare in China (Deng and Li 2020; Hu et al. 2020). Other international references included AI principles from Canada, Japan, Korea, Australia and the G20. Individual scholars’ works such as Nick Bostrom’s *Superintelligence: Paths, Dangers, Strategies*, Yuval Harari’s *Homo Deus: A Brief History of Tomorrow*, Herbert Marcuse’s *One-Dimensional Man* were also frequently cited. Some scholars were referred to for their general theories or entire body of work, for example, Immanuel Kant, Bruno Latour, and Luciano Floridi. Interviews with scholars such as Wendell Wallach and Alan Winfield also appeared in some articles (Fig. 2).

Unlike authors on WeChat, Zhihu users seldom refer to foreign sources. Academic users counted as exceptions for providing graphics or links to articles in English. However, few cited specific sources or provided bibliographic references. The few exceptions included John Brockman’s *25 possible ways to look at AI*, Lewis Mumford’s *The Myth of the Machine*, and *The Early History of Data Networks* by Gerard J. Holzmann and Bjoern Pehrson. Github content, scientific magazines such as *Nature* or *Science* and foreign news websites were also sometimes cited. More frequently, users made reference to science fiction such as *Matrix*, *Upgrade* and the series *Black Mirror*. These references were mostly used to address concerns related to AI, especially concerns over AI’s potential threat to humanity, which we elaborate on in Sect. 4.4.4.

To sum up, the discussions on AI ethics in China have been to some extent shaped by international deliberations. In line with our findings that the discussions were mainly driven by cultural elites on WeChat and the general public and individual tech professionals on Zhihu, the authors on WeChat also displayed more interests in and familiarity with international developments and debates, when compared to those on Zhihu. Moreover, the reference analysis demonstrated that deliberations in the USA and Europe have exerted great influence on the research of Chinese scholars and even tech companies, at least at the discursive level. Chinese researchers have extensively explored Western philosophies concerning science, technology and their social implications. They have, in turn, informed the Chinese public about global initiatives concerning the governance of AI. Although Zhihu users appeared to be less receptive to the international high-level deliberations on AI ethics and governance, they were tuned in to the imaginaries of AI created in science fiction produced in the US. These parallel engagements at the two levels – cultural elites and general public—with other parts of the world, although predominantly the Western world, provide a positive outlook for future societal dialogues on this topic at the international level.

### Context of discussion

**Fig. 3:**
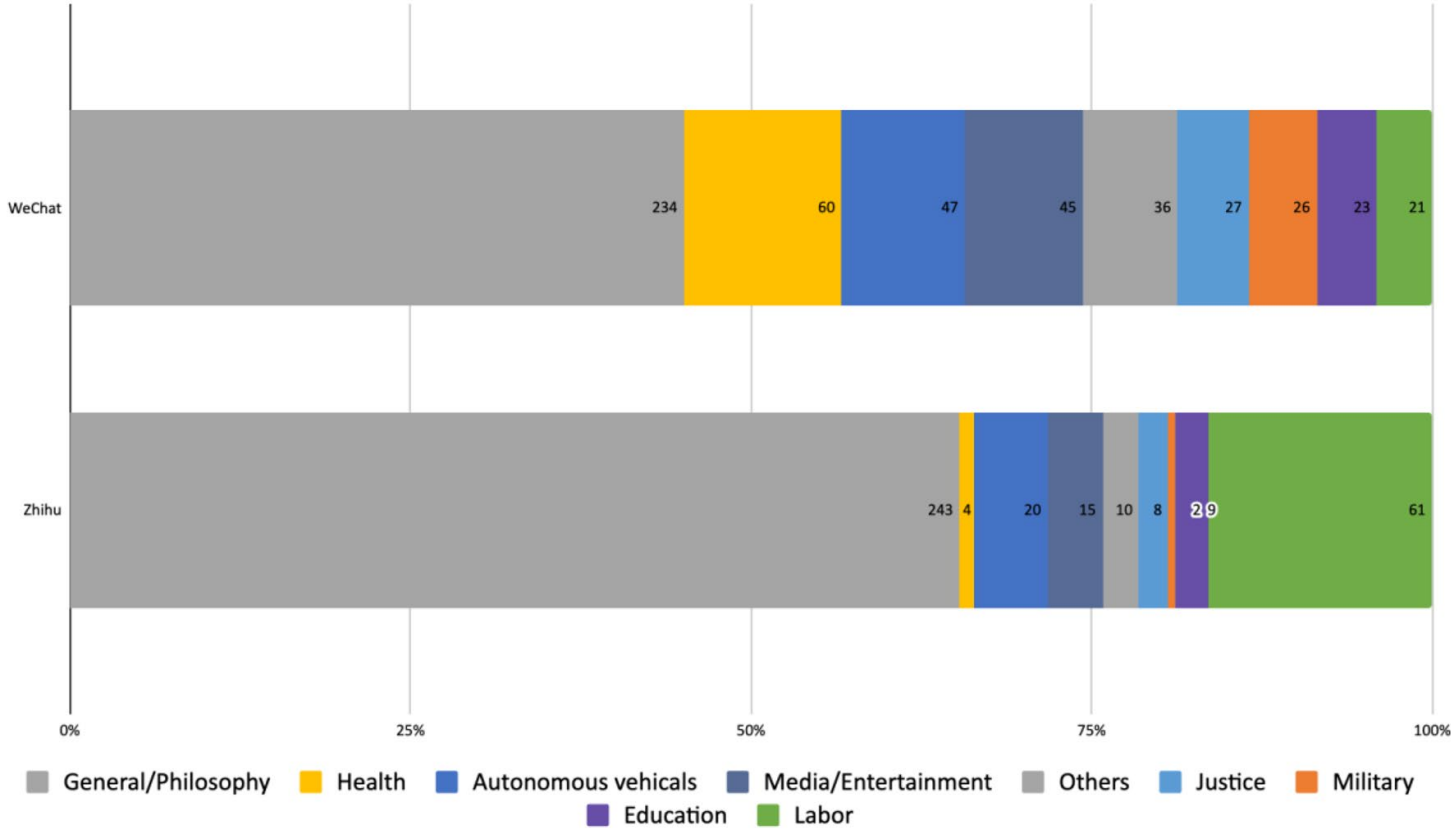
Context of discussion on WeChat and Zhihu

We coded the context(s) in which the WeChat articles and Zhihu posts discussed the ethical and social implications of AI. 45.1% of the articles on WeChat and 65.3% of the answers on Zhihu discussed the topic at an abstract level, approaching AI as a broad field and interrogating some fundamental philosophical questions. These discussions, which were coded under the ‘General/Philosophy’ category, can be summarized as covering, but not limited to the following aspects (Fig. 3):

1. The nature of technology itself (e.g., whether it can possess or develop morality and the ability to distinguish right from wrong).
2. The nature of humanity (e.g., consciousness, intelligence, creativity, senses, emotion) and what the use of AI means for this nature.
3. The relationship between humans and machines (e.g., love, competition), how they should interact and their responsibilities toward each other (e.g. no harm, mutual respect).
4. The changes in human societies caused by AI technologies (e.g., intelligence revolution or survival of the fittest), and the way in which people live and associate with each other.

In the discussions about AI applications in specific fields, on WeChat most were focused on healthcare (11.6%). Research on this topic seemed to have gained momentum in 2020, with a series of academic papers analyzing the ethical issues around the use of AI in assisted or automated diagnosis or prescription, AI medical monitoring wearables, AI-based health counseling apps, etc. (e.g., Zhang 2020, Zhou 2020, Chen and Zhang 2020; Zhou et al. 2020). The second most frequently addressed field of application was autonomous vehicles, which accounted for 9.1% of the discussions. Part of the reason was the use of the ‘trolley problem’ as a classical example to introduce the topic of AI ethics. 8.7% of the discussions on WeChat focused on AI in the media and other entertainment applications. Deepfake apps, Cambridge Analytica, and chat bots such as Microsoft’s Tay have triggered much media coverage on the potential harmful impacts of AI technologies. There were also a few articles investigating the ethical implications of using AI in journalism (e.g., Li 2019a; Zhao [Bibr CR80][Bibr CR81]). The remaining discussions addressed the use of AI in the judicial system (5.2%), autonomous weapons (5%), education (4.4%), the impact of AI on labor (4%), and miscellaneous other applications such as in financial and social services (6.9%).

On Zhihu, most posts discussed the ethical issues around AI in relation to labor (16.4%), such as whether or not AI will lead to more unemployment and/or human idleness. Negative consequences for individuals or for a specific group of people were frequently weighted against the overall “progress of society”, as many authors called it, which was generally seen more positively, and the “liberation of the people”, mentioned in several posts, from tedious labor to focus on more creative activities or even leisure in the long term. All other categories were only mentioned by a minority of users. ‘Autonomous driving’ (5.4%) was mostly addressed in relation to the ‘trolley problem’ or the latest developments by Chinese companies like Baidu in this technology. AI in the context of “media/entertainment” (4%) was mostly mentioned in relation to science fiction, chat bots such as Microsoft’s Tay (with regard to racist comments) and Little Ice (pornographic conversations).

Comparison of discussions on WeChat and Zhihu, despite the shared focus on general philosophical questions regarding human–machine relations, uncovered a clear difference with regards to AI applications across specific sectors. On WeChat, a recent surge of academic papers about AI in healthcare led this field to being the most discussed. However, the impact of AI on labor was paid the least attention to. Conversely, labor issues around AI attracted the most attention on Zhihu, and AI in healthcare the least. Zhihu general public users’ lack of interest in fields seemingly less relevant for them was demonstrated by the near absence of discussion on the use of AI in military scenarios.

### Perceptions of AI technologies

#### General assessment in terms of opportunity, risk and neutrality

The dataset for this research consisted of an analysis of opinion-based writing, meaning that much of the data contained assessments relating to the risks and/or opportunities that AI technologies posed to individuals as well as broader society. We coded the articles and posts mentioning risks and opportunities as ‘Neutral’ and those without such an assessment as ‘Not applicable.’ This section gives a quantitative overview of the presence of these assessments on both platforms. The detailed reasons given for these assessments are further analyzed in the Sects. 4.4.2–4.4.4.

On WeChat, most (37.7%) of the articles assessed AI technologies’ impact as neutral, acknowledging both the negatives and the positives. A significant number of the articles (28.1%) made no assessment at all, which were often academic papers that directly launched into an analysis of philosophical questions. 27.1% of the articles made the assessment that AI will bring risks, stressing AI technologies’ uncertain or unintended consequences. For example, Duan Weiwen, a researcher from the Chinese Academy of Social Sciences and the top expert on AI ethics in China, argued in an article that “The increasing application of AI and automated decision making systems including robots is not only an open-ended technological innovation with uncertain consequences, but also a social ethical experiment with a far-reaching impact in the history of human civilization” (Wen 2018). Only 7.1% of the articles assessed AI as generally beneficial.

On Zhihu, a slightly higher percentage of users (38.7%) focused on the risks of AI when compared to those who offered neutral assessments (35.5%). Risks were linked both to unintended consequences and non-foreseeable technological developments leading to AI acting in a “hostile” and “evil” way, or AI “overtaking” humans (He 2017). When compared to WeChat users, more (19.4%) of the Zhihu users made positive assessments of AI’s potential impacts. However, on Zhihu there were significantly fewer users (6.5%) who did not make any assessment at all.

#### Neutral perceptions

On both platforms, about one-third of the authors had neutral perceptions of AI’s impact on society at large or individuals. Authors on WeChat typically held the narrative that AI, similarly to other technologies, is a double-edged sword and its impacts would depend on how it would be used. This was particularly common among the academic, industry and media accounts, who often made a neutral assessment of AI in general at the beginning of the article, then discussed the risks and opportunities associated with the technology at length later on. For example, Tan Tieniu, a professor at the Institute of Automation of the Chinese Academy of Sciences, gave a keynote titled “Artificial Intelligence: Angels or Demons” which was posted by the WeChat public account “Towards Intelligence Forum”. In the keynote, he argued that,

High-tech itself makes no distinction between angels and demons, and so is AI. So, is the double-edged sword of AI an angel or a devil? It depends on human beings. We should plan ahead and form a joint force to ensure the full effect of AI in benefiting mankind! (Tan 2018).

In a WeChat article about its AI principles issued in 2018, Tencent Research Institute also claimed that “The birth of a new technology itself is not good or bad, but it is our responsibility to ensure that these technologies can become ‘good technologies’ through ethical norms, laws and various institutional designs” (Si 2018).

The neutral perceptions on Zhihu were mostly backed by the belief that humankind can control the direction in which AI will develop. This was often based on a distinction between weak and strong/super AI, which was commonly emphasized by Zhihu users who were familiar with the technologies. Some Zhihu users also argued that AI’s benefits and risks cannot be easily distinguished:

The first thing to be sure is that AI is absolutely beneficial to humans, but beneficial to humans as a whole does not mean that it is beneficial to every individual. The increase in automation in various industries will concentrate wealth in the hands of fewer and fewer people. Robots take jobs away from people, and although it improves overall productivity, do those people who have their jobs taken away have their real income increased? (Ma 2020)

Notably, some Zhihu users also saw this technological development as inevitable, expressing deterministic and transhumanist arguments. For example, one Zhihu user claimed that he was not afraid of AI because “what should come is always coming…most likely, if not the only, way for human beings to survive on the light-year scale and 100,000-year scale as civilization is to move from carbon-based to silicon-based. This [flesh-and-blood] body is still too fragile when facing certain situations” (Zhaotangmixiang 2014).

However, most WeChat articles and Zhihu posts’ neutral perceptions were derived from a combination of concerns and hopes associated with AI; a detailed analysis substantiating this assessment is in Sects. 4.4.3, which focuses on hopes and 4.4.4, which focuses on concerns.

#### Opportunities

On both platforms, only a small number of authors expressed their hopes for the opportunities that AI can bring to society or to individuals. Most of the hopes that were expressed fell into the following three categories: AI provides social benefits, humans and AI complement each other, and AI helps human society to revise and evolve.

In 7% of the WeChat articles and 11.6% of Zhihu posts authors expressed hopes about AI’s potential to provide social benefits. These included increasing productivity and efficiency, eradicating poverty, improving education, providing medical care, promoting sustainable development, relieving humans of tedious tasks, alleviating the shortage of labor in aging societies and increasing safety in autonomous cars and even weapons. Some also used specific AI applications to illustrate their points: for example, a system developed to prevent suicide by screening the Chinese social media platform Weibo for posts that show suicidal tendencies and dispatching rapid intervention rescue teams (Li 2020). Zhihu users also mentioned the benefits of algorithms in finding a suitable partner through assessing and matching various personality traits, and AI robots that cater to sexual needs (Winterhouse 2016).

2% of the articles on WeChat and 11.3% posts on Zhihu claimed that AI is beneficial because humans and AI complement each other. This argument typically holds that AI and humans can perform different tasks to different levels: humans are better at tasks involving creativity or emotions, and AI are better at more mechanical and repetitive work. One user used the field of education as an example: “I think AI can help improve students’ learning efficiency, while teachers’ repetitive teaching work will be gradually replaced, but there is no way for AI to really replace teachers…Education is education precisely because it is the awakening of personality and mind…And these things can only be achieved in the process of human interaction. Robots are certainly not able to transmit this kind of knowledge” (Yunduokecheng 2018).

1.4% of the articles on WeChat and 3.5% posts on Zhihu argued that AI can help human societies evolve by revising past rules and norms. This argument holds that the ethical norms in human society are not constant but need to be adapted to new environments as technology develops. For example, in an article published by Tencent Research Institute’s public account on WeChat, the author argued:

Smart assistants may become a powerful boost to our efforts to promote gender equality. We hope that the new technology will lead the whole society in a more equal and pluralistic direction, rather than cementing our existing biases through more preference algorithms (Wang [Bibr CR60][Bibr CR61]).

Many authors who held this type of opinion expressed belief in some kind of “new civilization” which would replace the older, inferior one (Fengqichanglin 2017). Lan Jiang, a philosophy professor at Nanjing University, analyzed brain–computer interface (BCI) technology and claimed in an article published by the party-state media outlet *Guangming Theory* that, “BCI will constitute a kind of transhumanism. Perhaps what we will see is not necessarily an ethical disaster, but a new kind of hope for ethics” (Lan 2019).

#### Concerns

**Fig. 4:**
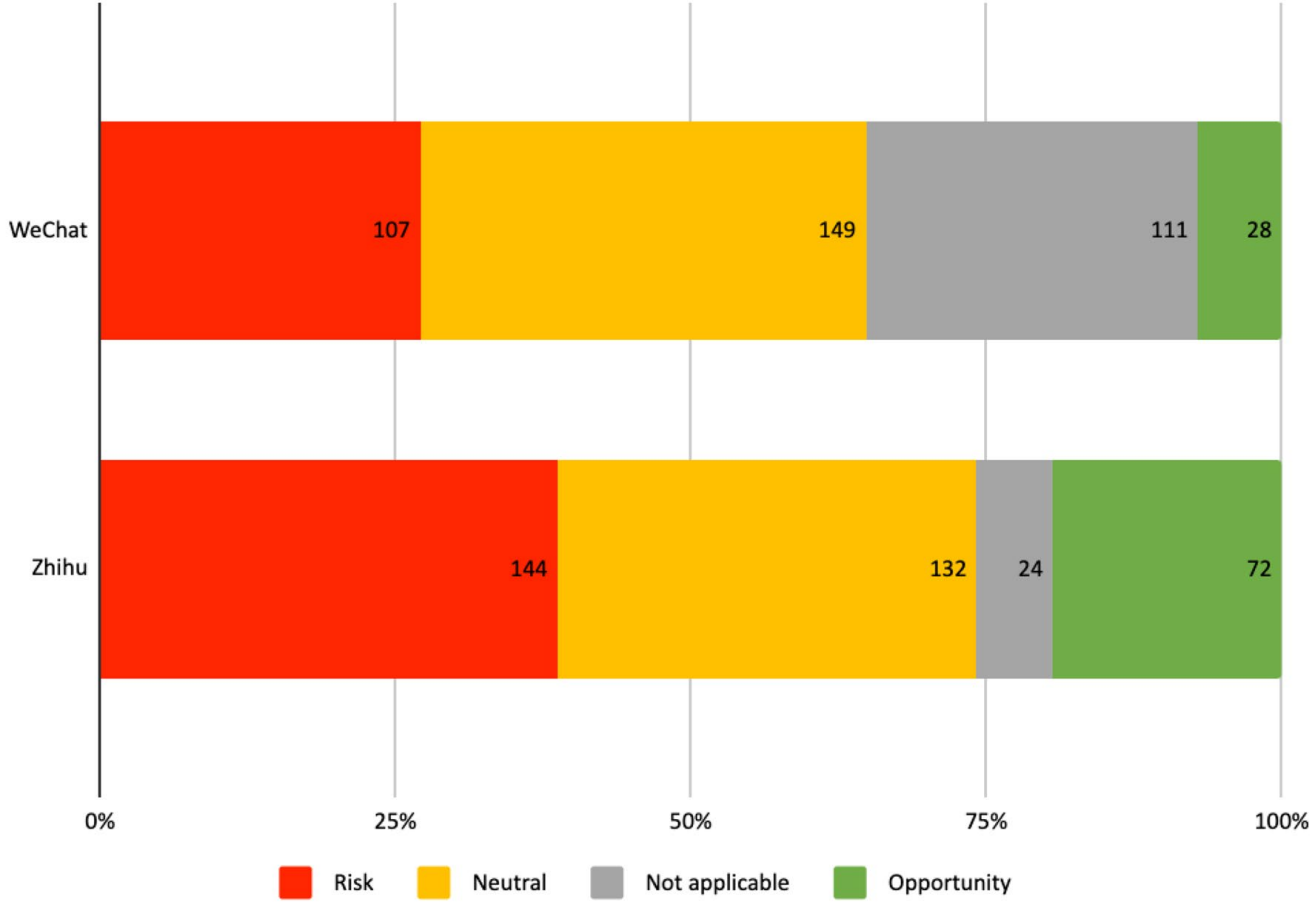
Opportunity/risk/neutral assessment of AI on WeChat and Zhihu

A significant share of the discussions on both platforms was devoted to concerns associated with the use of AI technologies. They largely fell into the following nine categories shown in Fig. 4. The following analysis focuses on the most frequent mentioned topics on the respective platforms.

9.1% of the concerns addressed on Zhihu and 10.3% on WeChat fell into the category ‘concerns for humanity.’ On the one hand, this included concerns inspired by science fiction scenarios about the threats AI could pose to the human race if AI were to surpass humans in terms of intelligence and capability (e.g., Luo 2015). This was particularly common among Zhihu users in the discussions prior to 2018. On the other hand, it included concerns regarding AI’s impacts on human nature, for example, that AI could become the “breakpoint” of human relations, leading to isolation, that it could replace or “technify” human activities, senses, body parts, and eventually humanity (Representative of Ethics Course 2020; Yang 2020). This type of concern was mostly addressed in the discussions on the general/philosophical level but sometimes in association with the use of AI in specific fields such as healthcare and education. Many authors expressed concern over the loss of the “human touch,” compassion and empathy in these scenarios, qualities which are deemed essential in caring and learning.

‘Responsibility concerns’ were the most frequently addressed concerns (18.1%) on WeChat. Discussions that fell under this category included those about AI systems’ moral standing (if AI can be regarded as having moral agency), their legal statuses/personalities, the social responsibility and liability when AI systems fail, and the intellectual property rights around AI.^8^ However, this concern was addressed by a small minority (4.1%) of Zhihu users, mostly linked to questions of liability with regard to autonomous driving.

16.1% of the concerns on WeChat addressed the potential bias embedded in AI systems or discriminative uses. However, most of the examples used to illustrate the harmful impacts of bias in AI systems—passed on from humans or as a result of a poor training dataset—are from abroad. Examples included Amazon’s recruiting algorithms favoring male applicants over females, and Google’s search engine that labels people with dark skin tones as apes (Informatization Collaborative Innovation Committee 2017). Examples from the Chinese local context for discriminative use of AI were only mentioned in the case of some commercial platforms’ use of algorithms for discriminatory pricing. On Zhihu, these concerns were rarely addressed (4.7%), and mostly in relation to Microsoft’s chatbots Tay and Little Ice. Users shared screenshots of conversations they had with those bots and their racist or sexist comments.

7.4% of the concerns on WeChat and 9.4% on Zhihu addressed issues relating to human autonomy and agency. Many authors on WeChat were worried about over-dependence on technologies, for example, if medical workers were to increasingly rely on AI for diagnosis and prescription it could lead to them losing relevant skills in the long run. Many authors also discussed the manipulative power of algorithms based on their access to users' behavior data and personal data. Cambridge Analytica was often cited as an example of this risk. On Zhihu, these concerns were frequently based on the possible emergence of strong AI with self-consciousness, which could manipulate humans.

Privacy was the second most frequently addressed concern on WeChat (16.5%), as the development of AI depends on large-scale collection of individuals’ personal and behavioral data. Notably, some scholars expressed pessimism towards the protection of privacy in relation to the introduction of AI. Liu Yibo, a scholar in the field of social governance, acknowledges that the boundaries of privacy will continue to shrink with the development of AI, arguing that in the future privacy will only exist at a conceptual level and humankind will enter the “post-privacy era” (Liu 2018). By contrast, only 2.9% of concerns expressed on Zhihu were about privacy. 8% of the articles on WeChat addressed concerns over AI’s impacts on employment. Despite being one of the least mentioned concerns on WeChat, this was the second most frequently mentioned concern on Zhihu (11.7%). While the view that the development of AI will inevitably lead to the elimination of jobs was prevalent, many authors disagreed as to how many new jobs might be created because of AI, and whether people should aim for less wage-based work and more space for personal development.

8.7% of the concerns on WeChat and 9.4% on Zhihu addressed issues regarding the increase of the digital divide and inequality. On WeChat, users mentioned inequality between social classes, regions, and industries arising from the uneven distribution of AI technologies. Du Junfei, a sociology professor, used two examples to illustrate his concerns: first, many senior citizens were excluded from social service because they were unable to use their health code during the COVID-19 pandemic; and second, the family of a 94-year-old woman had to carry her to the bank and maneuver her into an awkward position to use the facial recognition technology required by the banking services (Du 2020). Users on Zhihu stressed that AI is likely to increase societal inequality and even exploitation if AI remains in the hands of a few powerful actors for them to “squeeze the interests of the majority of people” (Yao 2019). Notably, Marxist theory appeared frequently in discussions on this platform. One user analyzed AI’s power in eliminating human labor using Marxist ideology, arguing that this would lead to the loss of peoples’ consumption power: “If capitalists still exist, then they will certainly prevent this from happening, specifically by demanding that…artificial intelligence will be limited to a level that is not too high or too low.” Two possible outcomes of AI development therefore depend on the existence of “capitalists”:

But if capitalists do not exist, or are eliminated, this would result in the means of production being transferred to universal ownership, and the manufactured products would be sufficient to satisfy the needs of so many people. The management body could be elected or be run directly by artificial intelligence...This does not mean that people would have completely given up work, but only that it would be the end of wage labor. Similarly, as at this point work is no longer wage labor, and you are no longer working for someone, you are doing it because you really want to (Yuexiashouwangzhe 2019).

AI systems’ technical complexity and perceived incomprehensibility—the ‘black box’ phenomenon—were the least mentioned concerns both on WeChat (5.6%) and on Zhihu (2.3%).

To summarize, the variety of concerns associated with the use of AI across social domains that were addressed on both platforms, and the different emphasis given to different issues, is noteworthy. On WeChat, where most authors were from academia, the media and industry, the majority of the concerns were related to individuals, such as responsibility, privacy, and bias. On Zhihu, where most of the authors were members of the general public, more emphasis was given to concerns at a society level, especially concerns for the future of humanity. This can be partly attributed to the general public’s fascination with science fiction; however, their concerns over employment, inequality and autonomy, which were not as prominently featured in the discussions on WeChat, showed how different social groups have different priorities when considering the ethical issues around AI. Together with the opportunities and neutral perceptions that were also expressed on the two platforms, this analysis provides a more complex picture of AI’s perception and development in China. The fervent pursuit of technological advancement by the state and companies, usually fueled on hopes for alleviating social issues, increasing efficiency and productivity, is accompanied by growing and diversifying concerns. One thing has become clear from these discussions: ensuing opportunities and risks associated with AI are not equally shared by the Chinese society, and adequate AI governance needs to be based on the negotiations of these hopes, concerns, and different interests.

### Recommendations for AI governance

**Fig. 5:**
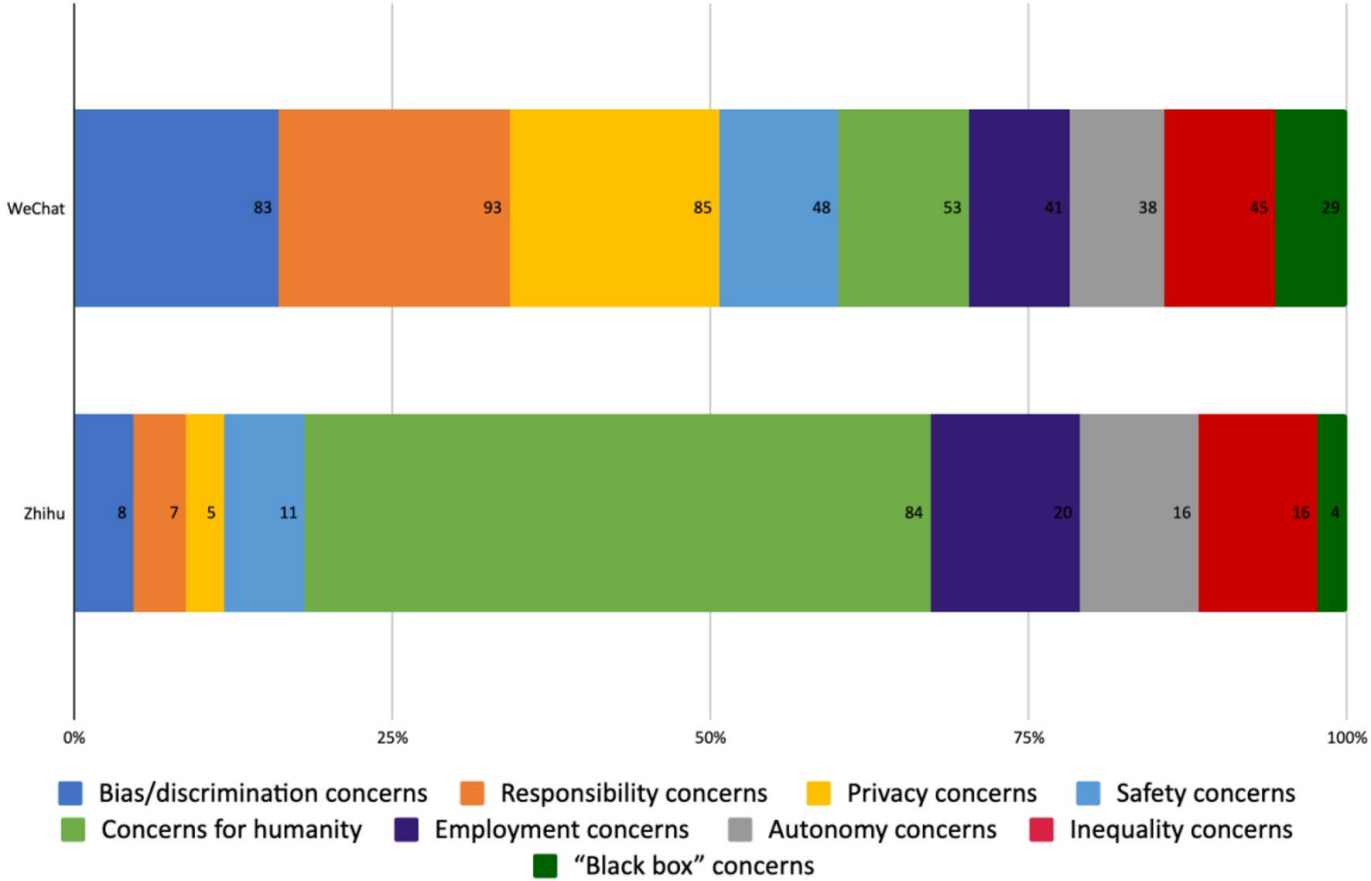
Types of concerns addressed on WeChat and Zhihu

63.5% of the articles on WeChat did not stop short at assessing the risk and benefits that AI can bring, but also gave recommendations on how to mitigate the risks and foster the opportunities associated with it. A significantly smaller number of posts on Zhihu did this (14.2%). The different recommendations largely fell into the eight categories shown in Fig. 5. Like in Sect. 4.4., the most frequently mentioned categories will be elaborated on further.

**Fig. 6:**
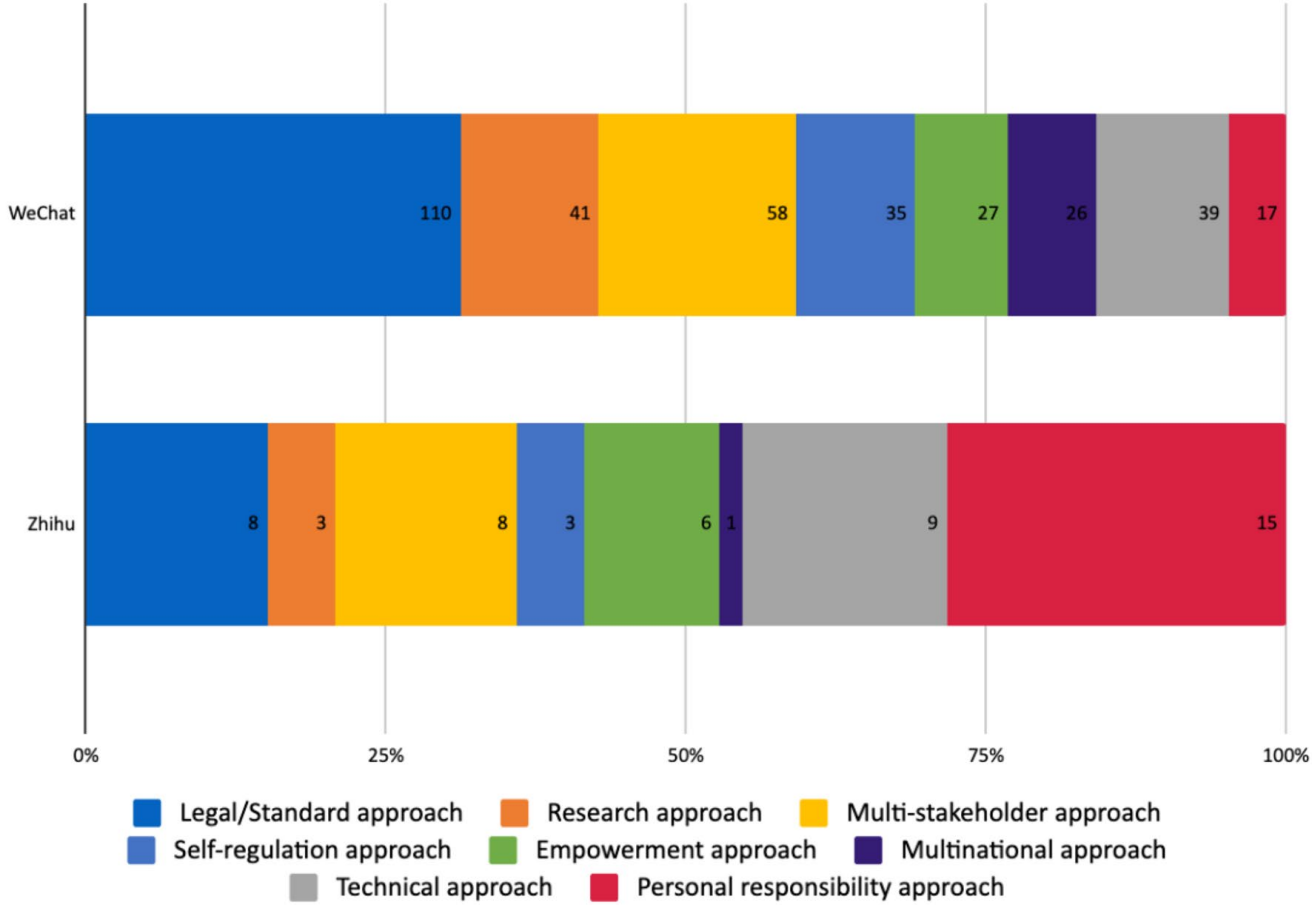
Types of recommendation offered on WeChat and Zhihu

Most of the recommendations (31.2%) on WeChat proposed the ‘legal/standard approach’, arguing that the state should be responsible for ensuring beneficial AI, and advocated for the establishment of regulations, laws, and standards. It also accounted for 15.1% of the recommendations from Zhihu authors (Fig. 6).

The ‘personal responsibility approach,’ advocating that individuals or humankind as a whole should be responsible for coping with the impacts of AI by improving themselves and learning to adapt to the future with AI, was the most recommended on Zhihu (28.3%) but least on WeChat (4.8%). A quote from a Zhihu author illustrated this type of argument well:

[Humans need to] maintain a tolerant mindset of continuous learning and updated cognition...Learn a bit of programming language properly, know yourself and your enemy, so you can never lose a battle.^9^ If you need to better understand AI, then learning how to manipulate it may be a necessary skill for the future (Bafangdejuzeng 2020).

Another Zhihu author attributed this responsibility to the whole of humankind:

Accepting the coming of the age of AI means adjusting how we ourselves evolve to new needs that will arise from new changes. Change is eternal; what doesn’t change is the moment (Qi 2019).

It is noteworthy that both the authors who advocated this approach echoed Chinese philosophical traditions. The first author cited Sun Tzu and the second reflected Daoist belief in eternal change. This approach, most often recommended by members of the general public on Zhihu, demonstrates that perceptions and responses towards AI can be culturally situated.

16.4% of recommendations on WeChat and 15.1% on Zhihu took the ‘multi-stakeholder approach’ which distributed the responsibility for the ethical development and use of AI across the shoulders of many. For example, scientists and engineers should be transparent and explain to the public the potential risks and uncertainties associated with the technologies. Managers of tech companies should be responsible for working with scientists, engineers and humanities scholars to conduct risk and ethical assessments. Policymakers should be responsible for science and technology policies, technological standards, regulations and laws that ensure the correct development trajectory. Humanities and social sciences scholars should research the social impacts of AI and formulate policy advice. The media should engage in promoting public understanding of AI. The public should also take responsibility for active participation in the social governance of AI. In addition, many authors stressed the importance of multidisciplinary research collaborations.

9.9% of recommendations on WeChat and 5.7% on Zhihu advocated for the ‘self-regulation approach’ which held that tech companies should be responsible for developing ethical AI. This was primarily because of the need to find a balance between ethics and innovation—to ensure technological development that brings societal benefits and economic growth while not stifling the innovation capabilities of enterprises. Suggested concrete measures for implementing self-regulation included the establishment of AI ethics committees composed of scientists, legal experts and project leaders, and engineers from within the companies. Besides committees, industry associations were sometimes advocated as the “external ethical gateway to control AI” (Chuangshi International Asset Management Group 2019). The Partnership on AI established by Google, Amazon.com, Microsoft Corporation, Facebook, IBM was also cited as a positive example of this. In addition, some authors on WeChat suggested the involvement of third-party review agencies to ensure compliance. Notably, but not surprisingly, this approach is mostly recommended by authors from the tech companies themselves (Tencent Research Institute 2018; Chuangshi International Asset Management Group 2019). It is the second least favored approach by the authors on Zhihu.

11% of the recommendations on WeChat and 17% on Zhihu supported the ‘technical approach’ which argued that ethical considerations should be part of the system design process. Ethical standards should be implemented by tangible techniques. For example, to tackle the ‘black box’ problem, technologies such as those employed by IBM Watson OpenScale, a tool to track and measure outcomes from AI models that helps ensure they are explicable and accurate, was cited for using technologies to solve technological shortcomings. Likewise, Zhihu users stressed the importance of ‘trial and error’ and a constant monitoring of mistakes/accidents in technology, either due to the technology itself or human behavior. In particular, one Zhihu user argued that ethical considerations should be involved in the process of designing AI, based on a specific order of ethical priority of a particular society (Brain 2015).

To summarize, among those who gave concrete recommendations for AI governance, there was again a clearly different emphasis between the cultural elites on WeChat and members of the general public on Zhihu. The former put more emphasis on the role of governments and the latter put more emphasis on people’s own responsibility. While authors on both platforms shared opinions in recommending the multi-stakeholder approach and technical approach, Zhihu authors demonstrated less trust in letting companies regulate themselves and their interest in international collaborations.

## Conclusion and implications

Our analysis has demonstrated that online discussions about the social and ethical implications of AI have been extremely lively and diverse in China. The discussions analyzed in this paper took place on different fora within a range of social groups, and had clearly varied foci and took different approaches. Despite similar assessments regarding the risks and opportunities associated with AI technologies, scholars, journalists, and leading tech corporations on the social media platform WeChat mainly addressed concerns over algorithmic bias, discriminative uses, responsibility and legal issues. They also demonstrated more familiarity with international scholarly and policy explorations. Members of the general public on the Zhihu platform, including those working in the tech industries, approached the issues around AI from an abstract level concerning the human collective in the long run. This was partly influenced by science fiction narratives produced in the US, but also concerns over job displacements caused by AI, rising inequality and the loss of autonomy. The cultural elites on WeChat were more vocal in terms of policy recommendations, stressing the responsibility of governments in regulations and leading multi-stakeholder participation. The recommendations given on Zhihu, however, emphasized the importance for individuals and human society to continue learning and adapting in order to remain competitive in the face of AI.

Above findings offer valuable ground for understanding the future trajectory of AI development in China. The diverse perceptions of AI in general and the wide range of concerns as identified in online discourse mean that the Chinese state’s and Chinese companies’ development of AI may continue, but not without addressing concerns raised by Chinese society. In fact, since 2020 multiple developments in China have been related to the concerns addressed in the above discussions. For example, the Chinese state started a crackdown on its domestic tech industry, launching investigations into how companies such as Alibaba and Tencent handled their users’ data and engaged in monopolistic and discriminatory business practices. In August 2021, China’s legislature passed the Personal Information Protection Law, called by some “the world’s strictest data-privacy law” (Xiao 2021). Although these developments put no constraints on how AI can be used by the government, the salience of concerns addressed to issues such as job displacement and increasing inequality among the members of the general public as identified on Zhihu will put increasing pressure on the government to manage the social implications of technological innovation.

Several implications for global dialogue on AI governance and directions for further research can be drawn from the findings in this paper which demonstrated both the influence of international developments in AI governance on and cultural specificities within the Chinese domestic discourse. First, although scholars, journalists and industry researchers in China appeared to be familiar with academic literature, especially in Europe and the US, regarding AI ethics, as well as policy developments globally, the ‘multinational approach’ did not top the approaches recommended on WeChat, even less on Zhihu. While China has shown a tepid attitude towards participating in international AI governance initiatives, such as signing onto G20’s non-binding AI principles and yet stayed away from Partnership in AI due to the fact that it consists of mostly Western actors, the lack of driving force in the Chinese public sphere is noteworthy and warrants further research. However, this does not mean that there is no prospect for international collaboration. Although observers outside China have anticipated that AI principles from China prioritize social responsibility and community relations over individual rights (Roberts et al. 2021), our research has demonstrated the importance of looking beyond official AI principles to the public discourse, which focused strongly on issues relevant to individuals such as algorithmic bias and privacy. Despite differences in philosophical traditions as well as political and economic priorities, the possibility of agreements on the practical implications of values such as security and privacy need to be examined through empirical evidence (Whittlestone et al. 2019). In addition, as demonstrated in this paper, the general public has expressed a sense of anxiety towards a future permeated with AI, in which their jobs and humanity could be threatened. This contradicts the widely held view that Chinese people demonstrate more positive attitudes toward digital technologies (e.g., The Digital Society Index 2018; Kostka 2019) and demonstrated similarities with the attitudes found in Western societies such as the UK (Cave et al. 2019). The reasons for Chinese society’s observed acceptance towards technologies may lie deeper in beliefs such as how to cope with changes and competition, as evident in some of the Zhihu authors’ recommendations. More research is needed to understand the thinking that underpins Chinese people’s attitudes towards AI technologies, especially in comparative perspectives. At a time of global competition and rivalry, especially between the Chinese party-state and liberal democracies, this research provides impetus for ongoing societal engagement, despite disagreements at government level.

## Data Availability

Available upon request.

## Footnotes

1 They are dubbed with this name because rumor has it that they are paid 50 cents RMB for each social media post they produce.

2 The two concepts are sometimes used interchangeably in modern Chinese discourse, although scholars have identified subtle differences between them in terms of semantic and cultural implications. For example, Chinese scholar Xinyu Yao claims that the etymology of “ethics” traces back to Western language and philosophy, embodying” reason, science and general will” while the etymology of “morality” traces back to Chinese language and philosophy, embodying “temperament, humanity and personal cultivation” (Yao 2006).

3 760 entries from Baidu and an additional 363 entries from Sogou were later added to the database.

4 The Zhihu platform puts the answer with the most replies or likes at the top of the page, ranking them first below the question. The date of the question is not displayed. The date of this first reply post is then captured via Baidu and Sogou as the first entry (consisting of question and answers) in the search results, while the same question might appear with the date of another answer further below in the search results. So it may be that the initial question related to the topic was posted on another date within the applied time frame. Then this post would be filed under the earliest date and duplicates would be removed from the overall sample.

5 We defined the categories for the type of author based on the availability of information, and the categories based on professional background. ‘Academia’, ‘Media’, ‘Industry’, ‘Government/Affiliated organizations’ proved to be the most workable criteria. If the author’s identity was given in a post, we coded the author category according to the author’s profession; if not, we coded it according to the type of public account. For example, if a scholar’s text was published in a media account, we coded it under the category of ‘Academia.’ Users classified as ‘General public’ are either anonymous, unverified authors, or authors with either no description or a prosaic one.

6 Due to limited capacity, we coded a maximum of three reasons, both concerns and hopes, for each unit of analysis (a post in the case of WeChat and an answer in the case of Zhihu).

7 Due to limited capacity, we coded a maximum of three recommendations.

8 Although the legal questions around AI do not strictly belong in the realm of AI ethics, due to their frequent mentions in the discussion we included them in our coding scheme.

9 The is quoted from Sun Tzu’s *The Art of War.* The Chinese original is “知己知彼, 百战不殆”.

## Code availability

Available upon request.
